# Use of Revised International Health Regulations during Influenza A (H1N1) Epidemic, 2009

**DOI:** 10.3201/eid1508.090665

**Published:** 2009-08

**Authors:** Rebecca Katz

**Affiliations:** The George Washington University, Washington DC, USA

**Keywords:** Influenza A virus, influenza, H1N1, viruses, pandemic, World Health Organization, international health regulations, expedited, podcast, perspective

## Abstract

Strong international health agreements and good planning created a structure and common procedure for nations involved in detection and evaluation of the emergence of influenza A (H1N1). This report describes a timeline of events that led to the determination of the epidemic as a public health emergency of international concern, following the agreed-upon procedures of the International Health Regulations. These events illustrate the need for sound international health agreements and should be a call to action for all nations to implement these agreements to the best of their abilities.

## Abstract 2

All nations should implement these regulations and cooperate in disease surveillance and data sharing.

In March 2009, human cases of infection with a novel strain of influenza A virus (H1N1) emerged in Mexico, the United States, and Canada. As of May 26, this contagious virus has spread to 46 countries, accounting for ≈13,000 cases. To date, >90 deaths caused by this virus have occurred, most of which have been in Mexico (*1*). Suspected cases are even more widespread, and the number of cases will inevitably continue to increase and the virus will spread to more countries in the coming weeks and months.

Predicting the course of the epidemic is difficult, but one can state with certainty that good multilateral plans and agreements facilitated the initial notification of the disease. Good planning has also enabled communication and action around the emerging epidemic in a manner that has been rational, predictable, and productive. These plans, which only came into being in the past 5 years, enabled an unprecedented level of timely cooperation and communication for assessing and responding to the novel influenza A virus (H1N1).

Some have argued that the initial detection of the outbreaks was delayed (*2*), and others have opined that the international disease surveillance and reporting system is severely crippled by a lack of resources (*3*). Although these debates will no doubt continue, it is crucial to document how, starting with initial notification by Mexico, the systems for communication and disease mitigation worked essentially as they were designed.

## Planning

### The International Health Regulations (2005)

A major international agreement, a regional agreement, and a multitude of pandemic plans put into place since 2005 have set the stage for the events of the past few weeks. In response to the threat of emerging infectious diseases, and pushed into action by the events related to the emergence of severe acute respiratory syndrome (SARS), the World Health Assembly agreed to accept the revised International Health Regulations in May 2005. These regulations, known as IHR (2005), are binding to all member states of the World Health Organization (WHO) and include several major provisions aimed at facilitating global communication and cooperation for early detection and containment of events termed public health emergencies of international concern (PHEIC). Although many international efforts in health have been disease specific, IHR (2005) focuses on the larger issues of ensuring competent surveillance and detection systems in every part of the world and a global commitment to work together to mitigate the consequences of a public health emergency.

Included in the regulations are provisions that member states are required to 1) establish a National IHR Focal Point for communication with WHO, 2) meet core capacity requirements for disease surveillance, 3) inform WHO in a timely fashion of any incident that might be considered a PHEIC, and 4) respond to additional requests for information by WHO (*4*). The revised regulations broadened the type of events that needed to be evaluated and reported to WHO to include a list of always notifiable diseases and an algorithm for determining a potential public health emergency, regardless of source or origin (*5*). In addition, the regulations clearly articulate that the purpose is to “prevent, protect against, control and provide a public health response to the international spread of disease” in a manner that “avoids unnecessary interference with international traffic and trade” (*6*).

The IHR (2005) were implemented in the summer of 2007. Two nations submitted reservations; the United States cited federalism concerns, and India clarified how it would regard regions infected with yellow fever (*7*). By the terms of the regulations, all member states should currently have in place a National IHR Focal Point for communication, should complete assessments of their disease surveillance capacity by the summer of 2009, and should develop and maintain their core surveillance and response capacities by the summer of 2012.

### Security and Prosperity Partnership of North America

In March 2005, the United States, Canada, and Mexico launched a trilateral agreement called the Security and Prosperity Partnership of North America (SPP). The purpose of this agreement was to enhance regional cooperation and information sharing around business competitiveness, energy, emergency management, securing of borders, and health (*8*). The health focus within SPP was to enhance public health cross-border coordination in infectious disease surveillance, prevention, and control. In particular, leaders of the 3 nations agreed to a set of principles that would guide collaboration in the detection and response to avian and pandemic influenza. These principles led to the formulation of the North American Plan for Avian and Pandemic Influenza (NAPAPI). This plan stresses the need for communication between nations and coordination in responding to the threat of a novel strain of influenza; it also lays out a set of actions for each nation relative to emergency coordination and communications, avian influenza, pandemic influenza, border monitoring and control measures, and critical infrastructure protection (*9*). A senior level coordinating body was established to facilitate planning and preparedness as well as to serve as a contact in the event of a human outbreak caused by a novel strain of influenza (*10*).

### Pandemic Plans

Spurred by fears of avian influenza (H5N1), the United States embarked on an aggressive policy to put into place a series of plans at the federal, state, and local levels. These pandemic plans address continuity of operations, social distancing strategies, vaccine and antiviral production and distribution, hospital surge capacity, and special considerations for vulnerable populations. In addition to plans, there were accompanying implementation schedules for implementing necessary infrastructure in place to ensure the plans would be useful should a pandemic emerge (*11*,*12*).

WHO has had a pandemic planning and guidance document available since 1999. In 2005, WHO revised the document in response to the threat of avian influenza. This document was revised and rereleased in April 2009, in part to reflect advances in global pandemic planning, the IHR (2005) entry into force, and scientific advances in the development and stockpiling of countermeasures (*13*).

## Events and IHR (2005)

I have outlined a series of events, beginning with the reporting by Mexico of an outbreak of acute respiratory illness. This event and subsequent events were linked with the corresponding article or provision in the IHR (2005), the SPP NAPAPI, or the WHO Pandemic Influenza Preparedness and Response guidance document. The events were organized according to the major goals of the IHR (2005): improving notification procedures, identifying public health emergencies of international concern, facilitating ongoing global communication during an emergency, and mitigating the consequences of the event through a coordinated response. In addition, the determination of pandemic phases as part of the IHR (2005) procedures, yet specific to this particular type of public health emergency, is discussed.

## Notification

On March 18, 2009, surveillance systems in Mexico alerted authorities to an unusual number of cases of influenza-like illness (*2*,*14*). After a few days of discussion starting on April 11 between the Pan American Health Organization (PAHO) and Mexican authorities regarding unusual numbers of acute respiratory infections, the authorities notified PAHO according to recommendations in IHR Focal Points of a potential PHEIC. The event was an outbreak of acute respiratory illness in the states of Veracruz and Oaxaca, Mexico (*15*,*16*).

On April 18, the United States, through the National IHR Focal Point, notified PAHO of 2 cases of human influenza A (H1N1) in children in San Diego County and Imperial County, California. The United States assessed that these cases could be a potential PHEIC (*17*).

The initial notification by Mexico and the United States of a potential PHEIC within their borders aligns with the following articles of the IHR (2005):

- IHR (2005) Article 4 (Responsible Authorities). Each state is responsible for designating a National IHR Focal Point for 24 × 7 × 365 communication with WHO, including for dissemination of information from WHO to relevant sectors of the state. These National IHR Focal Points were used to officially communicate the potential PHEICs to the regional WHO office (PAHO).
- IHR (2005) Annex 2 (Decision Instrument). The decision instrument in Annex 2 helps nations determine which events should be reported to the WHO as potential PHEICs. Mexico and the United States presumably used this decision instrument to determine if the events constituted a potential PHEIC.
- IHR (2005) Article 6 (Notification). State Parties shall notify WHO (through their WHO Regional Office–PAHO in this case) by way of the National IHR Focal Point of all events that may constitute a PHEIC. This notification must occur within 24 hours of assessment of the public health information by the national authority. After a notification, the State Party and WHO shall continue to communicate in a timely fashion about the notified event.

## Determination of a PHEIC

On April 25, the Director-General of WHO, after convening a meeting of the Emergency Committee, determined that the outbreak of novel influenza A (H1N1) constituted a PHEIC, and made a public announcement. This was the first declaration of a PHEIC after the entry into force of the IHR (2005) (*18*,*19*). The IHR Emergency Committee, which was convened by the Director-General on April 25, and which provides advice regarding the determination of the PHEIC, proposed that nations increase their active surveillance for unusual outbreaks of influenza-like illness (*20*).

The formation of the Emergency Committee and the process of declaring a PHEIC proceeded according to the following provisions of the IHR (2005):

- IHR (2005) Article 12 (Determination of a PHEIC). The Director-General determines on the basis of information received from the affected states whether an event constitutes a PHEIC. If the Director-General assesses the event to be a PHEIC, she then consults the affected states regarding her determination. Subsequently, the Director-General seeks the views of an Emergency Committee.
- IHR (2005) Article 48 (Emergency Committee: Terms of Reference and Composition) and Article 49 (Emergency Committee: Procedures). The Director-General establishes an Emergency Committee to provide views on whether an event constitutes a PHEIC; the termination of a PHEIC; and proposes issuance, modification, extension, or termination of temporary recommendations for mitigating the consequences of the PHEIC. The Emergency Committee may meet by teleconference, videoconference, or electronic communications.

## Ongoing Communication

After initial notification of the potential PHEICs by the United States and Mexico, WHO continued to maintain constant contact with the National IHR Focal Points. PAHO coordinated communication between the United States, Mexico, and Canada to better understand the emerging events (*14*,*21*).

National IHR Focal Points around the world continue to supply daily reporting of confirmed and suspected cases to WHO (*22*,*23*). WHO communicated with all member states through the National IHR Focal Points and the WHO public website to inform them of recommendations for actions to mitigate the consequences of the epidemic (*24*). On April 28, PAHO hosted a teleconference with health officials and ministers from 26 countries to exchange information on the influenza A (H1N1) epidemic (*25*).

The continued communication between the WHO and Member States, as well as between Mexico, Canada, and the United States, followed the regulations and provisions in the IHR (2005) and the SPP NAPAPI:

- IHR (2005) Article 6 (Notification). Following the initial notification, the State Party and WHO shall continue to communicate in a timely fashion about the notified event, including sharing updated detailed public health information on the notified event. This information includes case definitions, laboratory results, source and type of risk, and number of cases and deaths.
- IHR (2005) Article 11 (Provision of Information by WHO). WHO, in the most timely fashion possible, shall send information to all States Parties that enable the States to respond to the public health risk.
- IHR (2005). As part of IHR (2005), WHO developed a secure Event Information website to share timely information about public health events and emergencies among State Parties and WHO. This password-protected site is accessible to National IHR Focal Points.
- SPP NAPAPI. Chapter 2: Emergency Coordination and Communications. Mexico, Canada, and the United States agreed to share accurate and timely information before and during an outbreak. The 3 countries committed to working together so that all 3 nations use the same information to inform decision making and action.

## Coordinated Response

On April 25, a team of experts from PAHO arrived in Mexico to assist with the outbreak. The team comprised WHO experts from Geneva and Washington, DC, and experts from the US Centers for Disease Control and Prevention. The team supported the efforts of Mexico in the epidemiologic investigation, laboratory diagnosis, clinical management, communication, and outbreak management, and reported daily to WHO and PAHO (*14*,*26*).

WHO and PAHO arranged to have 489,000 treatments (treatment for an adult was 75-mg capsules, twice a day for 15 days) of oseltamivir shipped to Mexico and other countries in the Americas. Approximately 220,000 treatments of oseltamivir were shipped to 21 countries in the Americas from the United Nations Humanitarian Response Depot in Panama (*27*,*28*).

The Director-General of WHO, after receiving advice from the Emergency Committee, made temporary recommendations to support the mitigation of the epidemic. WHO did not recommend travel or trade restrictions related to the virus but did recommend that persons who were ill delay international travel and that persons in whom symptoms developed after international travel seek medical attention (*18*,*29*).

The United States and other countries with confirmed cases shared isolates and sequences of the influenza A virus (H1N1) with the international community in a timely fashion. Samples of the virus were shared for the purpose of risk assessment, analysis, and for making seed vaccine (*30*,*31*).

The role and responsibilities of WHO for coordinating and assisting in the global response to a public health emergency are outlined in the following provisions of the IHR (2005):

- IHR (2005) Article 15 (Temporary Recommendations). If a PHEIC has been declared, the Director-General shall issue temporary recommendations according to the procedure set out in Article 49 (Procedures for The Emergency Committee).
- IHR (2005) Article 13 (Public Health Response). At the request of a State Party, WHO will assist in the response to a public health emergency by providing technical guidance, assessing the effectiveness of control measures, and mobilizing international teams of experts to send to the affected area.

## Pandemic Phases

At the initial meeting of the Emergency Committee on April 25, members decided to maintain the current WHO-designated pandemic phase at a level 3 (no sustained human-to-human transmission sufficient to sustain community-level outbreaks) (*13*,*19*). The Emergency Committee met again on April 27, and on the basis of the developing epidemic, recommended changing from pandemic phase 3 to pandemic phase 4 (human-to-human transmission is verified). Following this recommendation, the Director-General upgraded the classification to pandemic phase 4 (*20*).

The epidemic continued to expand globally, and the Emergency Committee met again and determined that the pandemic classification should be changed from phase 4 to phase 5 (the same identified virus is causing sustained community-level outbreaks in multiple countries). The Director-General announced on April 29 that the world was at phase 5 on the WHO pandemic scale (*32*).

The meetings of the Emergency Committee followed the protocol of the IHR (2005) discussed above. As part of their recommendations for action, the committee cited the following excerpt from the WHO preparedness document:

- WHO Pandemic Influenza Preparedness and Response Section 3.2.2 (The Designation of the Global Pandemic Phase). Per the pandemic plan of WHO, the Director-General designated the global pandemic phase, consistent with the applicable provisions of the IHR (2005) and in consultation with affected Member States. Phase 4 signals sustained human-to-human transmission of the virus. Phase 5 indicates the virus is causing sustained outbreaks in >2 countries. Phase 5 suggests that a pandemic is imminent, although not a forgone conclusion.

## Actions Outside the Regulations

Although the global community generally adhered to the IHR (2005), supported WHO recommendations, and participated in unprecedented levels of information sharing, there are still areas in which nations may be withholding information or make unilateral decisions that do not support the language or spirit of the revised IHR. For example, certain countries recommended against travel to North America, although WHO did not issue such recommendations. Other nations interrupted trade of pork products from the United States, disregarding the determination by WHO and global scientists that cooked pork does not transmit the virus. In addition, some countries quarantined North American citizens, regardless of potential exposure to influenza A virus (H1N1). One of these countries defended its decision to quarantine persons from North America by citing what it believed is the failure of the United States and Mexico to implement entry and exit screening to detect cases of infection with influenza virus (H1N1) (*33*). However, the US government referred to WHO advisory and IHR Emergency Committee recommendations, which to date are not advising entry and exit screenings because WHO believes it would not help to reduce the spread of the disease (*29**,**34*).

In the past few weeks, WHO received reports of infection with influenza virus (H1N1) from many nations outside North America, several of which involved sizable numbers of cases. However, these countries claimed that their cases were linked to importation, with no sustained human-to-human transmission within their borders. Recent evidence in some nations of substantial increases in case counts makes sustained human-to-human transmission almost a certainty (which may lead WHO to raising the pandemic level to 6, per the WHO definition of pandemic phases). Further examination will be required to determine if nations were hesitant to admit such transmission or if previous cases were caused by importation (*35*).

## Discussion

The rapid succession of events in this timeline describing the first weeks of international communication and collaboration around the outbreak of a novel influenza A virus (H1N1) demonstrate the value of good planning and agreements for addressing public health emergencies. Creating solid structures and procedures for dealing with emergencies has been shown to be essential for an appropriate response and mitigation effort. Although it is impossible to predict the exact nature of an emergency, thoughtful planning enables all affected parties to know their responsibilities and to know with whom they need to work. Time is not wasted on developing procedures and contacts during an emergency. Instead, responders can focus on mitigating the consequences of the event. Planning does not guarantee that everything will run smoothly, or that all nations will adhere to agreed-upon regulations. However, the current situation suggests that mitigation outcomes and response efforts will be more successful than an outcome if plans and agreements did not exist.

Many of the provisions included in IHR (2005) came about as a result of WHO and global experience during the 2003 SARS epidemic. Comparing the experience of SARS with the current influenza (H1N1) event can serve as a means of measuring the usefulness of the regulations. An obvious comparison is the global communication mechanisms around an emerging epidemic. When WHO needed to reach the global community to alert it to the emergence of SARS, it needed to hold a press conference on a Saturday morning. No mechanism was in place for communicating with member states in a timely fashion. The creation of National IHR Focal Points enabled rapid communication between WHO and the entire global community, and guaranteed that proper authorities were notified and that information was shared with appropriate policy makers and responders. If only for this reason, the IHR (2005) can be deemed a success.

In this public health emergency, the revised IHR were used accurately and appropriately. The regulations were established in part to facilitate communication and formulate action in the identification of a PHEIC, and that is what happened. The SPP agreement was put into place to ensure regional cooperation in the event of a health emergency. Mexico, Canada, and the United States followed the SPP agreement and shared timely information. These events should serve as a call to action for each nation to do its best to fully implement IHR (2005) and engage in regional cooperation concerning disease surveillance and data sharing.
